# Multicenter assessment of the rapid Unyvero Blood Culture molecular assay

**DOI:** 10.1099/jmm.0.000804

**Published:** 2018-07-27

**Authors:** Sandra Christina Burrack-Lange, Yoann Personne, Monika Huber, Elisabeth Winkler, Jan Weile, Cornelius Knabbe, Julia Görig, Holger Rohde

**Affiliations:** ^1^​ Curetis GmbH, Max-Eyth-Straße 42, 71088, Holzgerlingen, Germany; ^2^​ SMZ Otto Wagner Spital, Pathologisch-Bakteriologisches Institut, Vienna, Austria; ^3^​ Institut für Laboratoriums- und Transfusionsmedizin, Herz- und Diabeteszentrum Nordrhein-Westfalen, Universitätsklinik der Ruhr-Universität Bochum, Bad Oeynhausen, Germany; ^4^​ Institut für Medizinische Mikrobiologie, Virologie und Hygiene, Universitätsklinikum Hamburg-Eppendorf, Martinistraße 52, 20246 Hamburg, Germany

**Keywords:** Bloodstream infection, sepsis, blood culture, diagnostics, antibiotic resistance, multiplex PCR

## Abstract

**Purpose:**

Bloodstream infections remain an important cause of morbidity and mortality. Rapid diagnosis can reduce the time from empiric antimicrobial therapy to targeted therapy and improve patient outcomes.

**Methodology:**

The fully automated Unyvero Blood Culture (BCU) Application (Curetis GmbH) can identify a broad panel of pathogens (36 analytes covering over 50 pathogens) and 16 antibiotic resistance gene markers simultaneously in about 5 h. The assay was evaluated in three clinical laboratories in comparison to routine microbiological procedures.

**Results:**

A total of 207 blood cultures were included in the study, and 90.5 % of the species identified by culture were covered by the Unyvero BCU panel with an overall sensitivity of 96.8 % and specificity of 99.8 %. The time to result was reduced on average by about 34 h. The assay accurately identified 95 % of the species, including 158/164 monomicrobial and 7/9 polymicrobial cultures. The Unyvero BCU Cartridge detected a large number of resistance markers including *mecA* (*n*=57), *aac(6*′*)aph(2*′′) (*n*=40), one *vanB* resistance gene, and six instances of *bla*_CTX-M_.

**Conclusion:**

The Unyvero BCU Application provided fast, reliable results, while significantly improving turnaround time in blood culture diagnostics.

## Introduction

Bloodstream infection is a serious clinical condition which can lead to sepsis, associated with a high rate of morbidity and mortality [1, 2]. Sepsis is the leading cause of death in intensive care units, and entails high healthcare-related costs worldwide [3–5]. Conventional microbiological methods for pathogen identification (ID) and susceptibility testing from blood cultures can take several days, resulting in empirical, often inappropriate, antimicrobial therapy that is continued for several days, before blood culture results are available. Reducing the time to pathogen and potential antibiotic resistance identification is crucial to ensure targeted antimicrobial therapy at an early stage, to improve patients’ outcomes, and lower the risk of mortality [6–10].

Various approaches for rapid identification of pathogens from positive blood culture bottles are available. These methods include real-time PCR, fluorescence *in situ* hybridization using peptide nucleic acid probes (PNA-FISH), PCR coupled to high-resolution melting curve analysis, and direct matrix-assisted laser desorption ionization–time of flight (MALDI-TOF) mass spectrometry [11–16]. However, these methods still require a certain amount of workload and technical skills, and have in some instances a narrow diagnostic spectrum. Additionally, none of them has the capacity to rapidly identify important antimicrobial susceptibility markers. New automated methods using multiplex PCR have been introduced in recent years to detect a panel of pathogens, but identify only a limited selection of resistance markers, or cover, respectively, only Gram-negative or Gram-positive bacteria [17, 18]. Rapid diagnostics assays, associated with antimicrobial stewardship programs and real-time feedback, have the potential to reduce the time to initiate the appropriate antimicrobial therapy [19–21].

The Unyvero System (Curetis GmbH) is a cartridge-based molecular diagnostic platform, for the detection of bacteria and fungi, as well as antibiotic resistance markers. The Unyvero Blood Culture (BCU) Application includes 36 analytes covering over 50 pathogens, and 16 antibiotic resistance gene markers for detection from positive blood culture bottles in around 5 h. The main objective of this prospective multicentre study was to assess the performance of the Unyvero BCU Application using blood culture bottles in comparison to routine microbiology testing.

## Methods

The study was conducted at laboratories of three tertiary-care hospitals in Europe: Otto-Wagner-Spital (OWS) in Vienna (Austria) from May to July 2016; Herz-und Diabeteszentrum NRW, Bad Oeynhausen (Germany) from April to June 2016; and Universitätsklinikum Hamburg-Eppendorf (UKE) (Germany) from June to August 2016.

Aerobic and anaerobic blood culture bottles were used from two different blood culture systems: Bactec (BD Diagnostic Systems) was used in Bad Oeynhausen and UKE while BacT/Alert (BioMérieux) was used in OWS. Recovery and selective culturing of yeasts and fungi was carried out with Bactec Mycosis IC/F bottles. If no microbial growth was detectable, incubation was terminated after 5 days. Bottles of the type Bactec Mycosis IC/F were incubated in the Bactec 9240 blood culture system, and BacT/Alert FA, -N and PF Plus used the incubation medium BacT/Alert 3D.

### Conventional microbiological methods

Positive blood culture bottles were subjected to Gram staining, and subcultures were streaked onto Columbia agar with 5 % sheep blood and Mac Conkey agar, Schaedler agar was used for anaerobic growth and Sabouraud agar for cultivation of fungi (all from Oxoid). After overnight growth, microorganisms were identified to species level using MALDI-TOF (Bruker Daltonics, Bremen, Germany) or biochemical profiling (Vitek2 XL, Biomerieux, Marcy L′Etolie, France). Susceptibility testing was carried out using a semi-automated system (Vitek 2, Biomerieux) or Kirby-Baur disc diffusion assays. Breakpoints to differentiate between susceptible and non-susceptible phenotypes were applied according to the European Committee on Antimicrobial Susceptibility Testing.

### Molecular pathogen identification using the Unyvero BCU Application

Once the blood culture bottle flagged positive, 500 µl fluid was removed from the bottle in a safety cabinet, 50 µl were added to the Unyvero B1 Sample Tube, which, after sealing, was placed into the Unyvero Lysator for processing (30 min). Subsequently, the sample tube was inserted with the Unyvero Master Mix (dNTPs, polymerase) into the Unyvero BCU Cartridge. Extraction, amplification, detection and analysis are automated within the cartridge. Assay results were considered valid, if the internal quality control gene was detected appropriately; otherwise, the assay was documented as invalid. A universal bacteria primer pair is used to detect bacteria not covered by species or genera-specific primers.

### Data analysis

A Unyvero BCU result was considered to be true positive (TP) or true negative (TN), if it corresponded to the reported culture result. In the case that conflicting results were obtained by conventional and molecular testing, discrepant result resolution was performed as follows. DNA was isolated from the sample using the Qiagen QIAamp DNA Blood Mini Kit; singleplex PCR was performed and PCR products sequenced bi-directionally, followed by blastn analysis. False positive (FP) Unyvero BCU results were changed to TP when PCR and sequencing were successful and BLASTn results confirmed the result reported by Unyvero. If the PCR failed, or if there was just a PCR amplification but no successful sequencing reaction, the Unyvero BCU result remained FP. False negative (FN) Unyvero results changed to TN when no PCR product was detectable. They remained FN when PCR and sequencing was successful, and the BLASTn results confirmed the result reported by microbiology, and when PCR was successful but amplicon sequencing failed.

### Calculations

Weighted sensitivity was calculated as 100×TP/(TP+FP). Weighted specificity was calculated as 100×TN/(FP+TN). Positive predictive values (PPV) were calculated as 100×TP/(TP+FP). Negative predictive values (NPV) were calculated as 100×TN/(FN+TN).

## Results

The performance of the Unyvero BCU Application was evaluated using a total of 207 blood cultures from 175 patients; 57 samples (47 patients) were tested at the OWS, 64 samples (55 patients) at Herz-und Diabeteszentrum NRW, Bad Oeynhausen, and 86 samples (73 patients) at the Universitätsklinikum Hamburg.

### Microbiology results

**Fig. 1.**
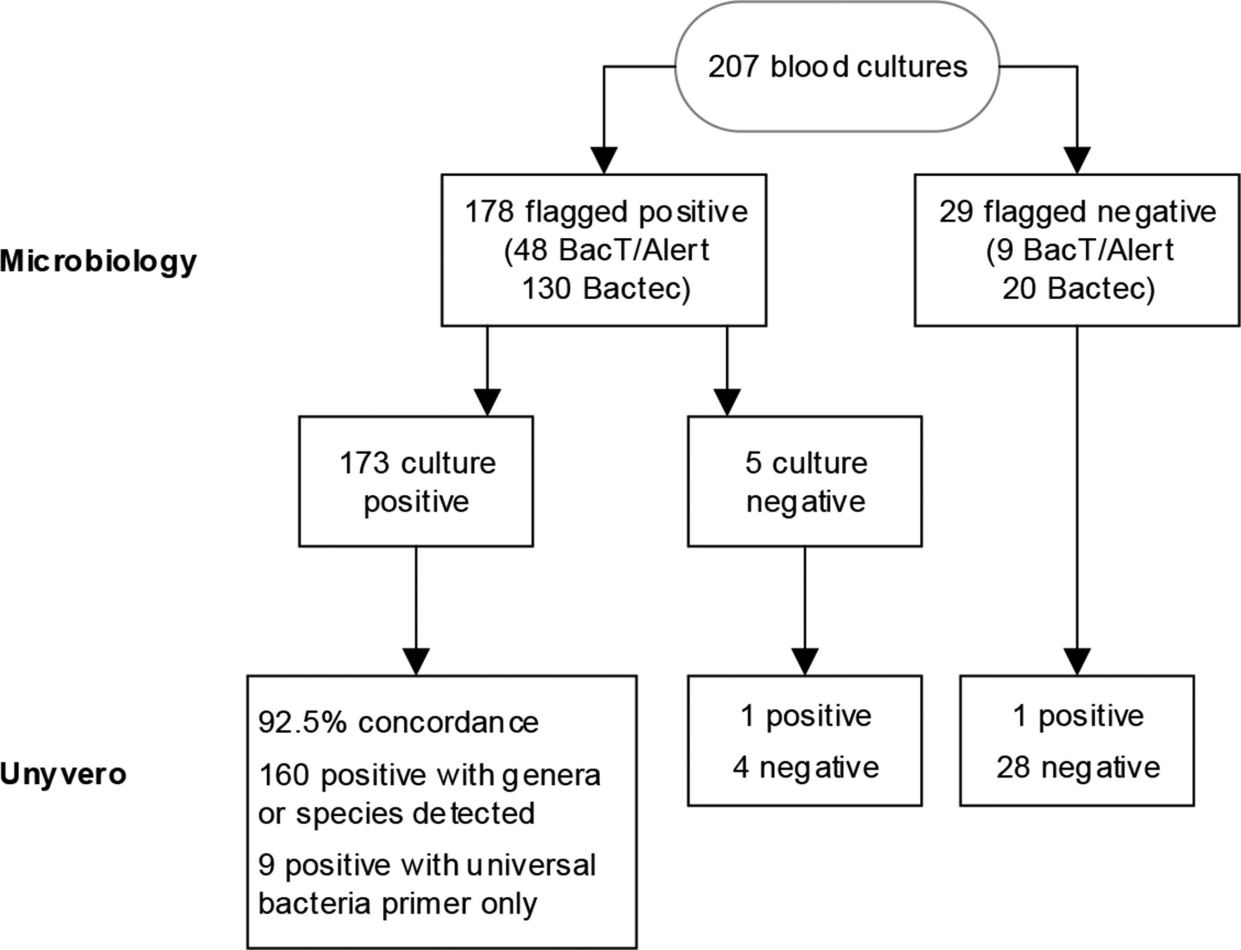
Flowchart of samples for routine microbiology cultures and Unyvero BCU Application.

Microorganisms were grown from 173 blood cultures (Fig. 1), the most prevalent microorganisms detected were coagulase negative staphylococci (CoNS) (*n*=77), *Escherichia coli* (*n*=22) and *Staphylococcus aureus* (*n*=16) (Table 1). No growth was obtained from 34 blood cultures, of which five were flagged positive in the BacT/Alert system (Gram staining was negative for two samples, positive for two and unknown for one sample). The BCU assay was also negative for all but two of these: one was positive for *Acinetobacter baumannii,* and one was positive with CoNS and *Propionibacterium acnes;* both samples were later confirmed positive by sequencing.

**Table 1.** Performance data of pathogens of the Unyvero BCU Application panel before discrepant result resolution

		TP	FP	TN	FN	Sensitivity	Specificity	PPV	NPV
**Total**						**95.0** %	**99.7** %	**89.1** %	**99.9** %
**Gram-negative bacteria**	**Total**	52	7	2839	0	*100* %	*99.75* %	*88.14* %	*100* %
	***Escherichia coli***	22	0	185	0	*100.0* %	*100.0* %	*100.0* %	*100.0* %
	***Klebsiella pneumoniae***	10	0	197	0	*100.0* %	*100.0* %	*100.0* %	*100.0* %
	***Klebsiella oxytoca***	2	1	204	0	*100.0* %	*99.5* %	*66.7* %	*100.0* %
	***Klebsiella variicola***	0	2	205	0	–	*99.0* %	*0.0* %	*100.0* %
	***Citrobacter freundii/koseri***	1	0	206	0	*100.0* %	*100.0* %	*100.0* %	*100.0* %
	***Proteus* spp.**	0	0	207	0	–	*100.0* %	–	*100.0* %
	***Acinetobacter baumannii* complex**	1	1	205	0	*100.0* %	*99.5* %	*50.0* %	*100.0* %
	***Pseudomonas aeruginosa***	6	0	201	0	*100.0* %	*100.0* %	*100.0* %	*100.0* %
	***Stenotrophomonas maltophilia***	1	1	205	0	*100.0* %	*99.5* %	*50.0* %	*100.0* %
	***Haemophilus influenzae***	0	0	207	0	–	*100.0* %	–	*100.0* %
	***Enterobacter cloacae* complex**	5	0	202	0	*100.0* %	*100.0* %	*100.0* %	*100.0* %
	***Enterobacter aerogenes***	2	1	204	0	*100.0* %	*99.5* %	*66.7* %	*100.0* %
	***Serratia marcescens***	2	0	205	0	*100.0* %	*100.0* %	*100.0* %	*100.0* %
	***Neisseria meningitidis***	0	1	206	0	–	*99.5* %	*0.0* %	*100.0* %
**Gram-positive bacteria**	**Total**	108	10	2362	4	*96* %	*99.58* %	*91.53* %	*100* %
	**Coagulase-negative staphylococci**	73	5	126	3	*96.1* %	*96.2* %	*93.6* %	*97.7* %
	***Staphylococcus aureus***	16	0	191	0	*100.0* %	*100.0* %	*100.0* %	*100.0* %
	***Streptococcus pneumoniae***	0	0	207	0	–	*100.0* %	–	*100.0* %
	***Enterococcus* spp.**	11	1	195	0	*100.0* %	*99.5* %	*91.7* %	*100.0* %
	including								
	*Enterococcus faecalis*	4	0	203	0	*100.0* %	*100.0* %	*100.0* %	*100.0* %
	***Streptococcus* spp.**	3	0	203	1	*75.0* %	*100.0* %	*100.0* %	*99.5* %
	***Streptococcus agalactiae***	0	0	207	0	–	*100.0* %	–	*100.0* %
	***Streptococcus pyogenes/dysgalactiae***	0	0	207	0	–	*100.0* %	–	*100.0* %
	***Listeria monocytogenes***	0	0	207	0	–	*100.0* %	–	*100.0* %
	***Corynebacterium* spp.**	0	0	207	0	–	*100.0* %	–	*100.0* %
	***Propionibacterium acnes***	1	4	202	0	*100.0* %	*98.1* %	*20.0* %	*100.0* %
	***Mycobacterium* spp.**	0	0	207	0	–	*100.0* %	–	*100.0* %
**Fungi**	**Total**	12	4	1635	5	*71* %	*99.76* %	*75.00* %	*100* %
	***Candida* spp. (incl. all Species+spp.)**	8	2	196	1	*88.9* %	*99.0* %	*80.0* %	*99.5* %
	including:								
	*Candida albicans*	1	1	205	0	*100.0* %	*99.5* %	*50.0* %	*100.0* %
	*Candida glabrata*	2	0	204	1	*66.7* %	*100.0* %	*100.0* %	*99.5* %
	*Candida parapsilosis*	1	0	204	2	*33.3* %	*100.0* %	*100.0* %	*99.0* %
	*Candida tropicalis*	0	0	206	1	*0.0* %	*100.0* %	–	*99.5* %
	*Candida dubliniensis*	0	0	207	0	–	*100.0* %	–	*100.0* %
	*Issatchenkia orientalis* (*Candida krusei*)	0	1	206	0	–	*99.5* %	*0.0* %	*100.0* %
	***Aspergillus* spp.**	0	0	207	0	–	*100.0* %	–	*100.0* %

### Panel coverage

In total, 160 of the 173 blood culture bottles with positive growth had microorganisms that were included in the Unyvero BCU Application as species or genera, covering 92.5 % of the clinical isolates during the study period. A total of 11 microorganisms were not part of the Unyvero BCU panel: *Alloiococcus otitis, Bacillus cereus, Bacteroides fragilis, Pseudomonas stutzeri, Lactobacillus rhamnosus, Raoutella ornitholytica, Raoutella planticola, Salmonella enterica, Saprochaete capitata, Staphylococcus schleiferi* (not included in the CoNS primers) and *Streptococcus mitis.* Nine of these were, however, detected by the included ‘universal bacteria’ primer. The two undetected species were *Bacteroides fragilis* and *Saprochaete capitate,* a fungal species not covered by the universal bacteria analyte.

### Performance data

Unyvero results were invalid in four samples (1.9 %), but three were successfully repeated.

The performance data for each of the microorganisms included in the Unyvero BCU Application was calculated before (Table 1) and after (Table 2) analysis of discrepant results. CoNS were identified as a group containing seven species of which *S. epidermidis* was found most often. The comparison with microbiology showed a total of 21 false positives (seven Gram-positive, ten Gram-negative and four fungi) and nine false negatives (four Gram-positive and five fungi).

**Table 2.** Performance data of pathogens of the Unyvero BCU Application panel after discrepant result resolution

		TP	FP	TN	FN	Sensitivity	Specificity	PPV	NPV
**Total**						**96.8** %	**99.8** %	**94.3** %	**99.9** %
**Gram-negative bacteria**	**Total**	53	6	2839	0	*100* %	*99.79* %	*89.83* %	*100* %
	***Escherichia coli***	22	0	185	0	*100.0* %	*100.0* %	*100.0* %	*100.0* %
	***Klebsiella pneumoniae***	10	0	197	0	*100.0* %	*100.0* %	*100.0* %	*100.0* %
	***Klebsiella oxytoca***	2	1	204	0	*100.0* %	*99.5* %	*66.7* %	*100.0* %
	***Klebsiella variicola***	0	2	205	0	–	*99.0* %	*0.0* %	*100.0* %
	***Citrobacter freundii/koseri***	1	0	206	0	*100.0* %	*100.0* %	*100.0* %	*100.0* %
	***Proteus* spp.**	0	0	207	0	–	*100.0* %	–	*100.0* %
	***Acinetobacter baumannii* complex**	2	0	205	0	*100.0* %	*100.0* %	*100.0* %	*100.0* %
	***Pseudomonas aeruginosa***	6	0	201	0	*100.0* %	*100.0* %	*100.0* %	*100.0* %
	***Stenotrophomonas maltophilia***	1	1	205	0	*100.0* %	*99.5* %	*50.0* %	*100.0* %
	***Haemophilus influenzae***	0	0	207	0	–	*100.0* %	–	*100.0* %
	***Enterobacter cloacae* complex**	5	0	202	0	*100.0* %	*100.0* %	*100.0* %	*100.0* %
	***Enterobacter aerogenes***	2	1	204	0	*100.0* %	*99.5* %	*66.7* %	*100.0* %
	***Serratia marcescens***	2	0	205	0	*100.0* %	*100.0* %	*100.0* %	*100.0* %
	***Neisseria meningitidis***	0	1	206	0	–	*99.5* %	*0.0* %	*100.0* %
**Gram-positive bacteria**	**Total**	115	3	2362	4	*97* %	*99.87* %	*97.46* %	*100* %
	**Coagulase-negative staphylococci**	77	1	126	3	*96.3* %	*99.2* %	*98.7* %	*97.7* %
	***Staphylococcus aureus***	16	0	191	0	*100.0* %	*100.0* %	*100.0* %	*100.0* %
	***Streptococcus pneumoniae***	0	0	207	0	–	*100.0* %	–	*100.0* %
	***Enterococcus* spp.**	11	1	195	0	*100.0* %	*99.5* %	*91.7* %	*100.0* %
	including								
	*Enterococcus faecalis*	4	0	203	0	*100.0* %	*100.0* %	*100.0* %	*100.0* %
	***Streptococcus* spp.**	3	0	203	1	*75.0* %	*100.0* %	*100.0* %	*99.5* %
	***Streptococcus agalactiae***	0	0	207	0	–	*100.0* %	–	*100.0* %
	***Streptococcus pyogenes/dysgalactiae***	0	0	207	0	–	*100.0* %	–	*100.0* %
	***Listeria monocytogenes***	0	0	207	0	–	*100.0* %	–	*100.0* %
	***Corynebacterium* spp.**	0	0	207	0	–	*100.0* %	–	*100.0* %
	***Propionibacterium acnes***	4	1	202	0	*100.0* %	*99.5* %	*80.0* %	*100.0* %
	***Mycobacterium* spp.**	0	0	207	0	–	*100.0* %	–	*100.0* %
**Fungi**	**Total**	13	2	1228	2	*87* %	*99.84* %	*86.67* %	*100* %
	***Candida* spp. (incl. all Species+spp.)**	8	1	198	0	*100.0* %	*99.5* %	*88.9* %	*100.0* %
	including:								
	*Candida albicans*	2	0	205	0	*100.0* %	*100.0* %	*100.0* %	*100.0* %
	*Candida glabrata*	2	0	205	0	*100.0* %	*100.0* %	*100.0* %	*100.0* %
	*Candida parapsilosis*	1	0		1	*50.0* %	–	*100.0* %	*0.0* %
	*Candida tropicalis*	0	0		1	*0.0* %	–	–	*0.0* %
	*Candida dubliniensis*	0	0	207	0	–	*100.0* %	–	*100.0* %
	*Issatchenkia orientalis* (*Candida krusei*)	0	1	206	0	–	*99.5* %	*0.0* %	*100.0* %
	***Aspergillus* spp.**	0	0	207	0	–	*100.0* %	–	*100.0* %

In 13 samples, Unyvero BCU detected additional microorganisms compared to conventional culture methods: five CoNS, four *Propionibacterium acnes* (two in polymicrobial growth), two *Klebsiella variicola* (in polymicrobial growth), as well as one *Acinetobacter baumannii*, *Klebsiella oxytoca* (in polymicrobial growth), *Enterobacter aerogenes*, *Neisseria meningitidis, Stenotrophomonas maltophila* (in polymicrobial growth), *Enterococcus* spp.*, Candida kruseii* and *Candida albicans*.

### Discrepant result analysis

Following discrepant result resolution with singleplex PCR and sequencing, ten previously considered false positive results were identified as being true positive: four CoNS, three *P. acnes*, one *A. baumannii*, one *C. albicans* and one *Candida* spp. Overall weighted sensitivity and specificity values were calculated at 96.8 and 99.8%, respectively; with 87 % sensitivity for fungi, 100 % for Gram-negative and 97 % for Gram-positive bacteria (Table 2).

The Unyvero BCU Application correctly identified 197/207 samples (95 %), including 158/164 samples with monomicrobial growth. We identified nine samples with polymicrobial growth (Table 3), two of them contained *B. cereus,* which is not included in the panel. In the remaining samples, the Unyvero BCU was able to detect the multiple microorganisms in all but one sample, where it failed to detect the CoNS in one sample containing *Klebsiella pneumoniae* and *Staphylococcus haemolyticus,* and in one sample *P. acnes* was also detected as a false positive in addition to the other two species detected correctly.

**Table 3.** Comparison of pathogens found in Unyvero BCU Application and routine microbiology results of polymicrobial infections

Routine microbiology	Unyvero BCU	Comments
*Enterococcus faecium; Enterococcus faecalis*	*Enterococcus* spp*.; Enterococcus faecalis*	
*Staphylococcus epidermidis; Enterococcus faecium*	Coagulase negative staphylococci; *Enterococcus* spp.	
*Bacillus cereus; Streptococcus mitis*	Universal bacteria	*B. cereus* not present in panel
*Bacillus cereus*; *Streptococcus mitis*	*Streptococcus* spp.	*B. cereus* not present in panel
*Klebsiella pneumoniae; Staphylococcus haemolyticus*	*Klebsiella pneumoniae*	
*S. epidermidis; Pseudomonas aeruginosa*	CoNS; *Pseudomonas aeruginosa; Propionibacterium acnes*	*P. acnes* false positive
*Serratia marcescens; Enterococcus faecalis*	*Serratia marcescens; Enterococcus faecalis*	
*Enterobacter cloacae; Staphylococcus epidermidis*	*Enterob. cloacae complex;* CoNS	
*S.aureus; Staphylococcus epidermidis*	*S. aureus;* CoNS	

In addition, the Unyvero BCU assay correctly identified an additional five polymicrobial infections where three *P. acnes*, one CoNS and one *C. albicans* were missed by culture, whereas five other polymicrobial infections could not be confirmed with additional PCR and sequencing.

### Detection of antibiotic resistance gene

In total, 119 resistance genes were detected with the Unyvero BCU Application with *mecA* (*n*=57), *aac(6′)aph(2′′)* (*n*=40), *ermA* (*n*=10), *bla*_CTX-M_ (*n*=6) and *aacA4* (*n*=5) the most prevalent markers in various combinations, and one instance of *vanB*. Except for two MRSA, all other *mecA* genes found originated from CoNS. As expected, all *bla*_CTX-M_ were detected exclusively with *Enterobacteriacea*e (*E. coli*, *n*=2; *K. pneumoniae*, *n*=4); and *vanB* was detected with *Enterococcus* sp. Although the Unyvero BCU covers eight genes for carbapenem resistance, no microorganism was found to be positive for carbapenem resistance during the study period.

Complete antimicrobial susceptibility testing (AST) results were only available for a subset of 121 samples. Unyvero results with detection of resistance markers associated with confirmed cases of phenotypic resistances are listed in Table 4. Due to the low number of detections, positive and negative predictive values could only be calculated for some of the resistance markers (Table 5).

**Table 4.** Detections of antibiotic resistance markers by Unyvero BCU Application in comparison to culture in a subgroup of 121 samples

Antibiotic substance class	Resistance marker on Unyvero BCU panel	No. of detections with BCU	No. of detections with confirmed phenotypic resistance
Third generation Cephalosporins	*bla*_ctx-M_	1	1
Carbapenems	*bla*_kpc_, *bla*_imp_, *bla*_ndm_, *bla*_oxa-23_, *bla*_oxa 24-40_, *bla*_oxa-48_, *bla*_oxa-58_, *bla*_vim_	0	0
Oxacillin	*mecA, mecC*	29	26
Macrolide/Lincosamide	*ermA*	5	1
Vancomycin	*vanA, vanB*	0	0
Aminoglycosides	*aacA4*	3	3
	*aac(6′)aph(2′′)*	24	12

**Table 5.** PPV and NPV for selected antibiotic resistance markers detected by Unyvero BCU

Resistance marker	TP	TN	FP	FN	PPV	NPV
*mecA*	26	19	3	1	89.7 %	95.0 %
*aacA4*	3	11	0	2	100.0 %	84.6 %
*aac(6′)/aph(2′′)*	12	29	12	0	50.0 %	100.0 %
*ermA*	1	38	4	6	20.0 %	86.4 %

### Time to results

Overall, the average time to detection (TTD) for positive blood cultures with conventional plating methods was 24 : 38 h for pathogen identification (data available from 94 samples), and 47 : 54 h for full ID and AST results (data available from 158 samples). In comparison, the average TTD with the Unyvero BCU was 13 : 24 h, even though testing time from sample in to result was around 4.5 h.

## Discussion

Bloodstream infections are a common cause of morbidity and mortality in hospitalized patients, which new technologies that are able to provide rapid pathogen and resistance marker identification from blood cultures aim to reduce. Here, we describe for the first time the performance of the rapid and easy to use Unyvero BCU Application in a multicenter routine diagnostic setting.

We found a good correlation between the assay and routine culture methods with a total sensitivity of 96.8 % and specificity of 99.8 %. All identified Gram-negative bacteria (*n*=53) were detected while 4/115 Gram-positive and 2/13 fungi were not identified by the assay. Overall, 13 organisms were not included on the panel, the presence of the ‘universal bacteria’ analyte allowed for the detection of 11 of these, which is therefore a useful means to confirm false positive results.

Molecular diagnostic methods relying on DNA amplification could suffer from the composition of blood cultures, which varies between manufacturers. In this study, we tested four different blood culture bottle types from two manufacturers, and obtained similar performance results for all (data not shown).

Current standard laboratory methods rely on Gram staining followed by bacterial cultures with complete results available in 2 days or more. During the study period, the pathogen and resistance marker identification were available approximately 34 h faster using the Unyvero BCU Application when compared to culture-based diagnostics. It is to note, because there is no need for an initial Gram staining and the workflow is fast and easy, turnaround time could be further decreased, if the system were to be used as near as possible to the patient.

Increasing rates of antimicrobial-resistant pathogens prompt empirical use of broad-spectrum antibiotics, and inappropriate initial antimicrobial therapy for septic shock occurs in about 20 % of patients [22]. Although not assessed here, the rapid detection, or lack of, resistance genes could result in faster adaptation of targeted antimicrobial therapy, which is expected to improve patient outcomes, reduce mortality and impact length of hospital stays [22, 23]. It is to note that the Unyvero BCU Application identified 57 *mecA* cases, 45 cases of potential resistance to aminoglycosides (40× *aac(6′)aph(2′′)*, 5× *aacA4*), one *vanB* resistance gene, and six instances of *bla*_CTX-M_. No carbapenem-resistant isolates were identified during our study. In a subset of samples for which AST results were available, we found a good correlation between the presence of resistance markers and phenotypic resistance, except for aminoglycoside resistance, where the presence of *aac(6′)aph(2′′)* only correlated with resistance to gentamycin in 50 % of the cases.

Short-term subculture or direct-from-broth pathogen identification methods, using MALDI-TOF mass spectrometry [24, 25] have been described to deliver results faster than conventional plating methods, but these are unable to provide information on antimicrobial resistance markers and to identify multiple organisms. Detection of polymicrobial infections is critical, since mortality in these patients is significantly higher compared to monomicrobial infections, and empiric treatment is inadequate in over 50 % of cases [26, 27]. We only obtained nine samples with polymicrobial infections (4.2 %) here, which is below the rates usually described [28–30]. Seven samples had pathogens present in the panel and six of these were identified. The assay was able to correctly detect five samples with an additional pathogen that had not been reported, bringing our polymicrobial detection rate to 6.7 %. The Unyvero BCU Application, therefore, seems to be adequate to detect polymicrobial infections; a larger number of samples is, however, necessary for a firm conclusion.

Despite using over 200 positive blood culture bottles, we obtained a low number of certain organisms, while others could not be detected at all. The presence of these rare organisms such as *L. monocytogenes*, *S. pneumoniae* and *N. meningitidis* is clinically significant, which makes their inclusion in the panel adequate. Nevertheless, an optimal analysis of system performance would require a larger subset of samples, which was a limiting factor in this study.

The system has quick hands on time but, ideally, time to results (4 h 30) could be further improved. Running costs compared to routine methods, not assessed here, should also be carefully considered before implementation in each institution.

In conclusion, the Unyvero BCU assay shows a high degree of accuracy for pathogen identification, and it has significantly faster identification times. This may have a meaningful impact on patient care, which should be further assessed in additional interventional studies which should also calculate the cost effectiveness of the technology.
